# Identification of preschool children's physical fitness phenotypes and their association with multidimensional body shape indicators: a data-driven approach with risk prediction modeling

**DOI:** 10.3389/fpubh.2026.1773424

**Published:** 2026-03-02

**Authors:** Xiaoxiao Chen, Deqiang Zhao, Aoyu Zhang, Xiang Pan, Chuanmiao Wang, Jiaxin Chen, Yanfeng Zhang

**Affiliations:** 1 School of Physical Education and Sports Rehabilitation, Jinzhou Medical University, Jinzhou, Liaoning, China; 2 China Institute of Sport Science, Beijing, China; 3 School of Medicine, Tianshi College of Tianjin, Tianjin, China; 4 Graduate School of Health and Sports Science, Juntendo University, Inzai, Japan

**Keywords:** body shape indicators, cluster analysis, physical fitness phenotypes, preschool children, risk prediction

## Abstract

**Objective:**

Traditional physical fitness assessments in young children often rely on single indicators, which fail to capture integrated ability patterns. The common use of body mass index (BMI) to link morphology and fitness has limited discriminant validity. This study aimed to identify naturally emerging physical fitness profiles among preschool children in Macao using a data-driven approach and to develop a screening tool based on multi-dimensional body shape indicators—beyond BMI—for identifying children at risk of low physical fitness.

**Methods:**

The sample comprised 3,180 children aged 3–6 years from the Macao China Physical Fitness Surveillance and Physical Activity Survey (2010, 2015, 2020). K-means clustering was performed on six standardized physical fitness test scores to identify intrinsic fitness profiles. One-way ANOVA was used to compare body shape indicators across clusters. Using the low-fitness cluster as the dependent variable, logistic regression identified key morphological predictors, from which a composite discriminant index was constructed. Predictive performance was evaluated using receiver operating characteristic (ROC) curve analysis.

**Results:**

Cluster analysis revealed three distinct physical fitness profiles: low, moderate, and high fitness. ANOVA showed significant gradient differences across clusters for all body shape indicators (height, weight, circumferences, etc.). Effect sizes (Cohen's f) ranged from 0.025 to 0.190. Logistic regression identified four core predictors: height, weight, waist circumference, and pelvic width. The composite body shape index demonstrated good discriminative ability for low fitness risk, with an area under the curve (AUC) of 0.779 (95% CI: 0.742–0.800). At the optimal cut-off, sensitivity was 77.7%, and specificity was 66.8%. The index showed consistent performance in sex-stratified analyses (Boys: AUC = 0.87; Girls: AUC = 0.88).

**Conclusion:**

This study confirms that preschool children's physical fitness develops in distinct, internally coherent profiles, closely associated with body morphology. The composite index based on waist circumference and pelvic width—among other measures—proves more effective than BMI alone in identifying children at risk of low fitness. It offers a practical tool for rapid, early screening during routine health checks and supports targeted physical activity interventions.

## 1 Introduction

The preschool period (3–6 years) is a critical window for the development of fundamental motor skills and physical growth. Fitness levels during this stage not only influence current health, activity participation, and quality of life but also have long-term implications for physical conditioning, habitual physical activity, cognitive ability, school readiness, and social development (1–3). Establishing a scientific, valid, and forward-looking physical fitness assessment system for young children is therefore a central issue in early health promotion and holistic development, with significant implications for precise intervention and population health.

Current fitness assessments typically rely on isolated performance measures (4–6). While useful for evaluating specific motor skills, such approaches fail to capture the multidimensional, synergistic, and heterogeneous nature of overall physical fitness and cannot systematically address the fundamental question of what typical integrated fitness development patterns exist among children (7). This limits holistic understanding and hinders personalized guidance. In parallel, BMI remains the most widely used morphological indicator internationally due to its simplicity. However, its validity is particularly challenged in children aged 3–6 years, a period of rapid and non-uniform changes in body composition (8). Numerous studies note that BMI cannot distinguish between lean mass and fat mass or reflect fat distribution (e.g., central adiposity) (9–11). Evidence suggests that body composition (muscle-to-fat ratio) and fat distribution may be more closely linked to motor competence and metabolic health than overall adiposity (12, 13). Thus, relying solely on BMI provides a fragmented and limited link between morphology and comprehensive physical ability, offering insufficient basis for differentiated health promotion and fitness intervention (14).

To address these gaps, prior research has often correlated single fitness tests with isolated anthropometrics or focused on long-term growth indices. However, a significant methodological gap exists in objectively identifying holistic fitness phenotypes from multi-test data and subsequently linking these phenotypes to a practical, multidimensional morphological screening tool suitable for immediate risk assessment. This study aims to fill this gap. Specifically, this study aimed to: (1) identify naturally emerging physical fitness phenotypes among preschool children in Macao using data-driven clustering; (2) examine the association between these phenotypes and multidimensional body shape indicators; (3) develop and validate a composite body shape index for screening children at risk of low physical fitness. The application of unsupervised machine learning (cluster analysis) represents a substantive methodological advance by shifting the analytical focus from comparing children on isolated tests to identifying their inherent, integrated ability patterns. This approach is grounded in the recognition that physical fitness is a latent construct best revealed through pattern recognition in multi-dimensional data.

## 2 Methods

### 2.1 Participants

Data were drawn from the Macao China Physical Fitness Surveillance and Physical Activity Survey (2010, 2015, 2020), which used stratified random cluster sampling with one-year age groups (15). The study included 3,180 children aged 3–6 years. Parents or guardians provided written informed consent, and the study was approved by the Ethics Committee of the China Institute of Sport Science (CISS-2019-06-07). While combining data from three waves increases sample size and generalizability within the Macao context, we acknowledge the potential for unmeasured cohort or period effects as a study limitation. Data collection followed standardized national protocols with trained examiners to ensure consistency, although formal inter-rater reliability metrics were not recorded for all waves.

### 2.2 Measures

Height and weight were measured following the China Physical Fitness Standards for Preschool Children (CPFS-preschool) manual, and BMI was calculated. Physical fitness was assessed using six tests: standing long jump, tennis ball throw, 10-m shuttle run, 15-m obstacle run, sit-and-reach, and balance beam walk, administered according to CPFS-preschool guidelines (16). Raw scores were standardized (Z-scores) by age and sex. All raw scores were converted to age- and sex-specific Z-scores based on the entire combined sample to control for developmental differences prior to clustering, aligning with our aim to identify fitness patterns independent of normal age/sex variation.

### 2.3 Data analysis

#### 2.3.1 Clustering analysis.

To identify latent fitness profiles, K-means clustering was performed on the six standardized fitness Z-scores. The choice of K-means was justified by its efficiency with large samples, its common use for phenotype discovery, and the standardized, continuous nature of our variables. We addressed key assumptions and stability: (1) variables were standardized to equalize scales; (2) the Euclidean distance metric was used; (3) the algorithm was run with 50 random initializations to minimize the impact of initial centroid placement and achieve a stable solution; (4) cluster stability was assessed by calculating the average silhouette width (0.41), indicating fair structure. The optimal number of clusters (k = 3) was determined using the elbow method and scree plot of within-cluster sum of squares. To support the suitability of the data for structure detection, we report the Kaiser-Meyer-Olkin (KMO) measure of sampling adequacy (0.82) and Bartlett's test of sphericity (*p* < 0.001). We clarify that these tests indicate the data's suitability for multivariate analysis (like PCA used for visualization) rather than directly validating the cluster solution.

#### 2.3.2 Group comparisons.

One-way ANOVA was used to compare body shape indicators across the three clusters. Assumptions of homogeneity of variance and normality were tested using Levene's test and Shapiro-Wilk test on residuals, respectively. For indicators where Levene's test was significant (*p* < 0.05), Welch's ANOVA was applied, and this is specified in the results. Effect sizes are reported using Cohen's f. *Post-hoc* comparisons used Tukey's HSD test.

#### 2.3.3 Predictive modeling.

Logistic regression was performed with the low-fitness cluster as the dependent variable (1 = low, 0 = moderate/high) and all body shape indicators as potential predictors. Model building followed a purposeful selection approach. Key demographic covariates (age, sex, survey year) were examined. Age and sex were not included as their effects were already statistically controlled for via Z-score standardization of the fitness outcomes used to define the dependent variable. Including them again in the prediction model could lead to over-adjustment. Survey year was tested as a covariate but was not a significant predictor (*p* = 0.43) and did not improve model fit (Likelihood Ratio Test *p* = 0.41), thus it was excluded for parsimony. Multicollinearity was assessed using variance inflation factors (VIF < 5). A composite Body Shape Index was derived from the final model's coefficients.

#### 2.3.4 Validation and comparison

The index's discriminative performance was evaluated using ROC curve analysis. The area under the curve (AUC) with 95% confidence interval (derived from 1,000 bootstrap samples) was reported. Sensitivity and specificity at the optimal cut-off (Youden's index) were calculated. Internal validity was assessed via 10-fold cross-validation, reporting the mean cross-validated AUC. To formally test the claimed superiority over BMI, a separate logistic regression model using only BMI as a predictor was fitted, and its ROC curve was compared to the composite index's ROC curve using DeLong's test for two correlated ROC curves. Additionally, analyses were stratified by sex to explore potential differences, with ROC curves reported for boys and girls separately. All analyses used α = 0.05 and were performed in R (version 4.3.1).

## 3 Results

### 3.1 Sample characteristics

The final sample included 3,180 children (1,903 boys, 1,277 girls) across three survey years (Table 1).

**Table 1:** Sample distribution by year, age, and sex.

**Year**	**Age**	**Boys**	**Girls**	**Total**
	3	136	72	208
2010	4	173	108	281
	5	175	98	273
	6	95	73	168
	3	142	91	233
	4	170	119	289
2015	5	205	139	344
	6	89	82	171
	3	209	132	341
2020	4	189	125	314
	5	222	160	382
	6	98	78	176
Total		1,903	1,277	3,180

### 3.2 Identification of physical fitness phenotypes

**Figure 1:**
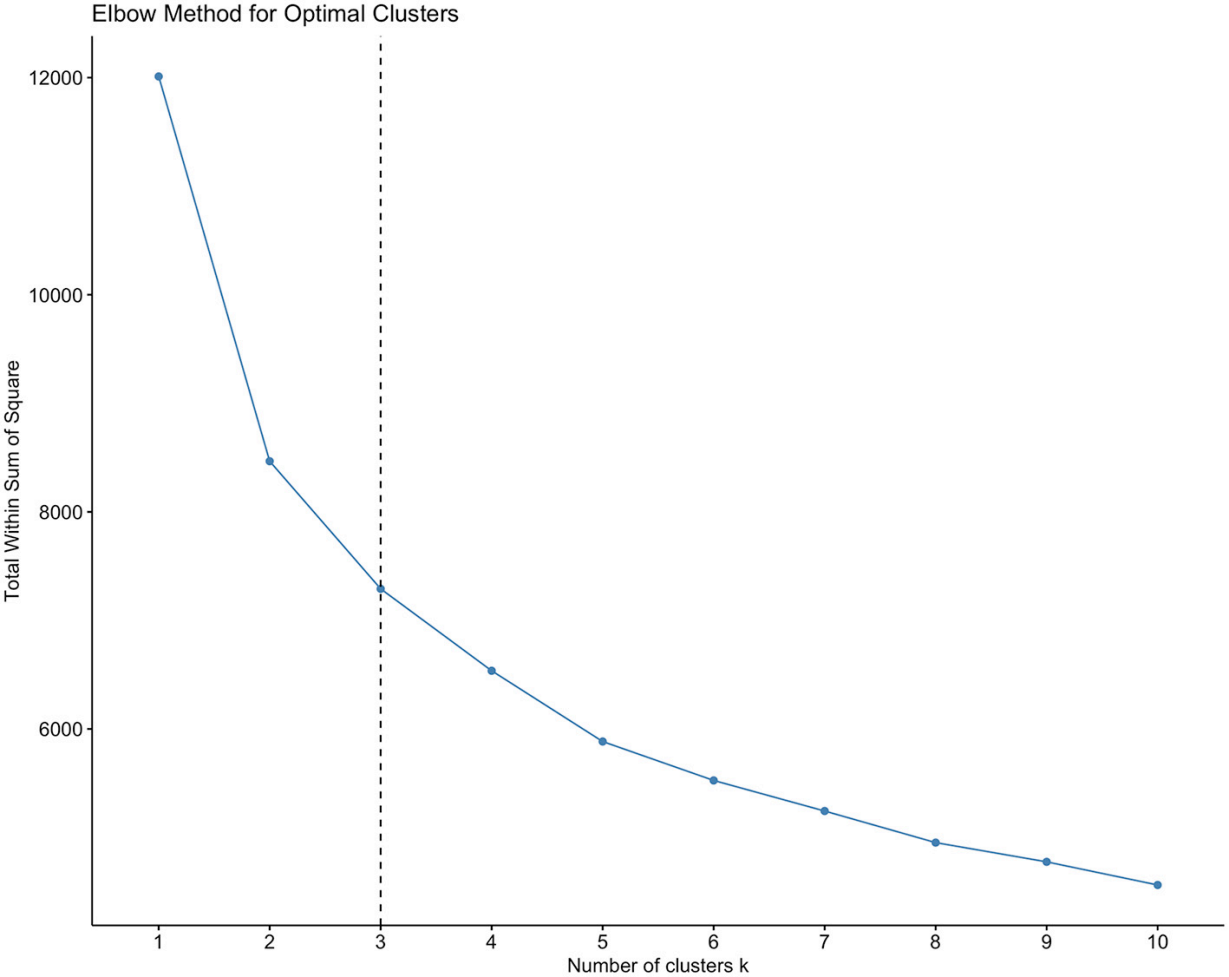
Elbow method for optimal clusters.

**Figure 2:**
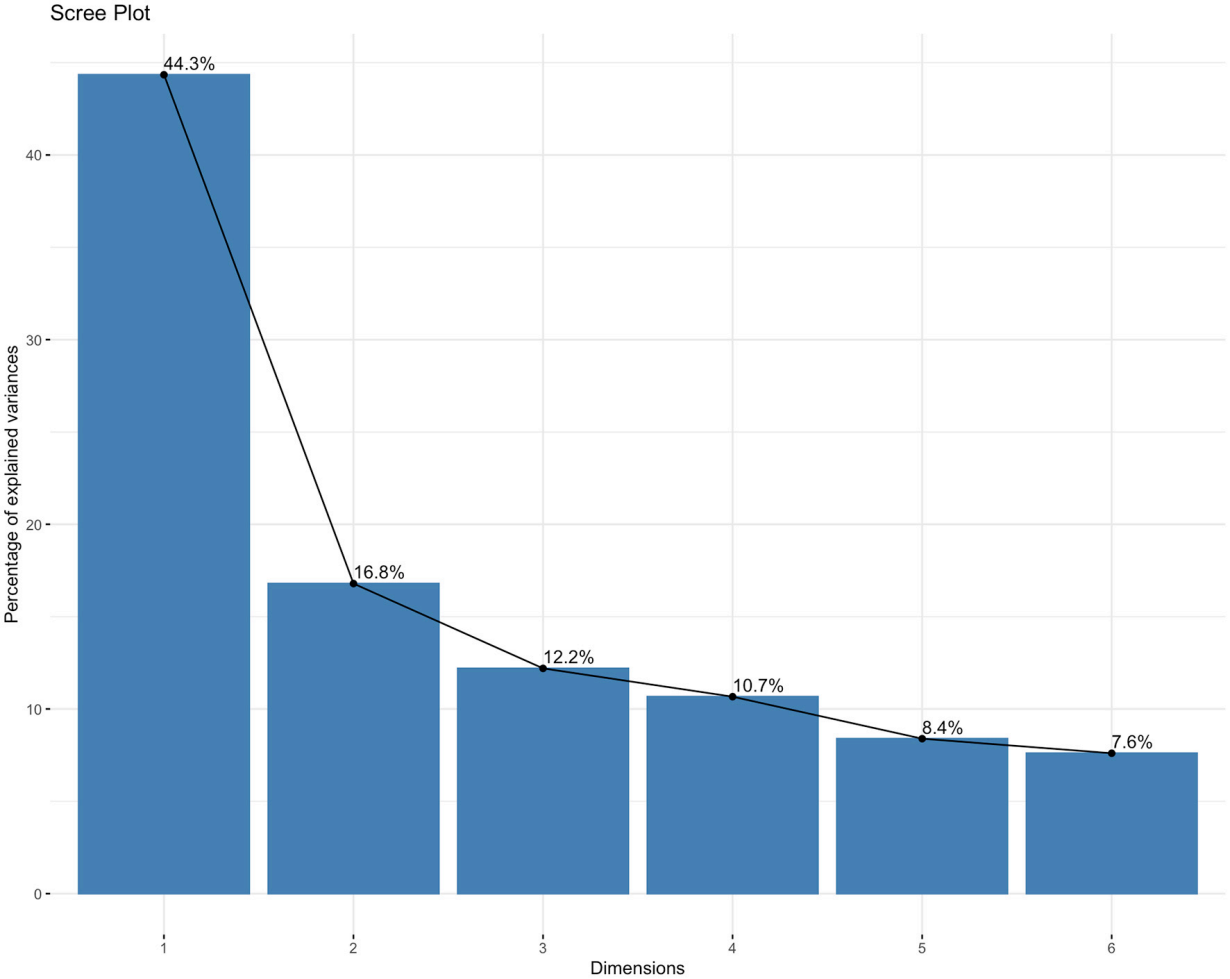
Scree plot.

**Figure 3:**
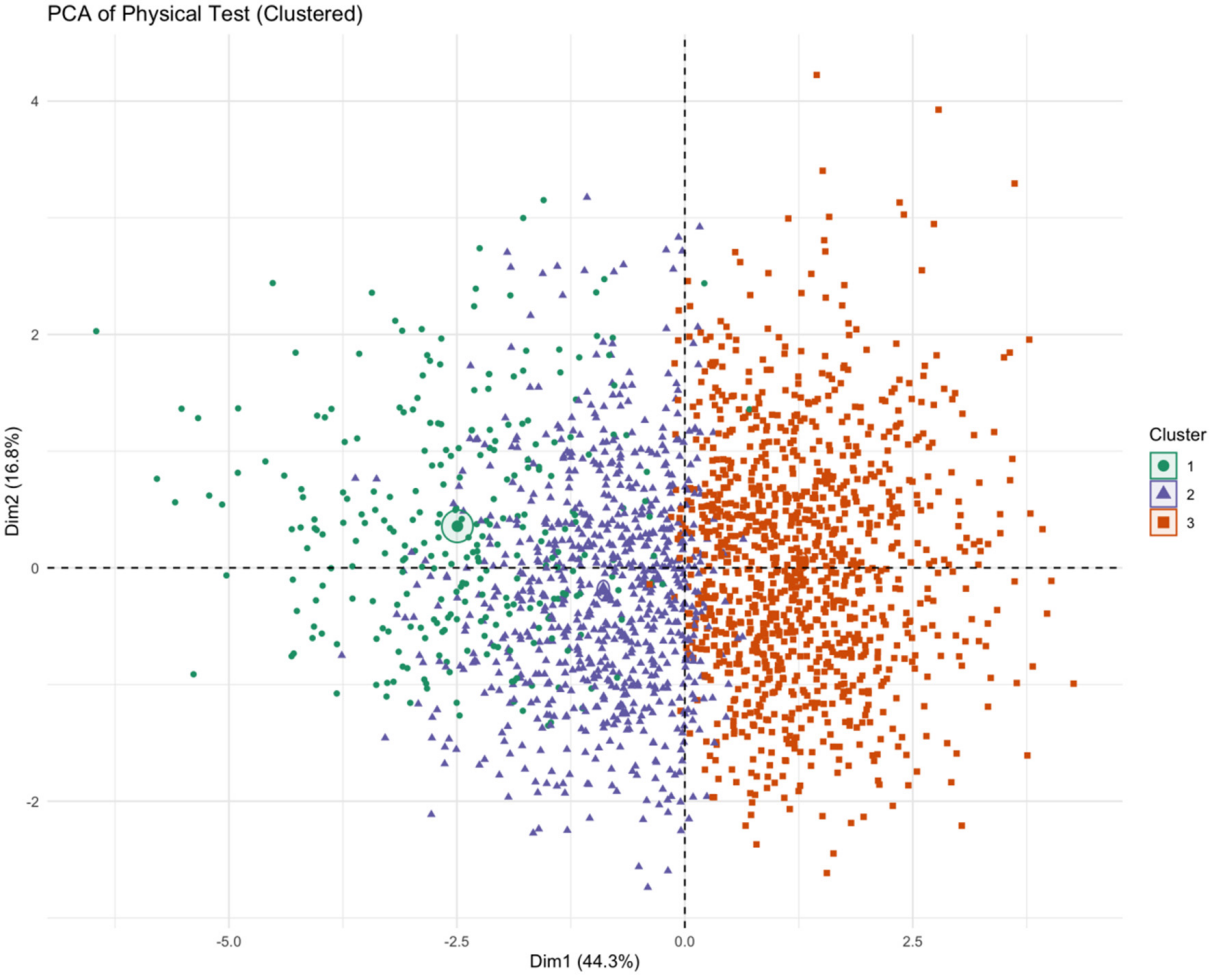
PCA biplot.

**Figure 4:**
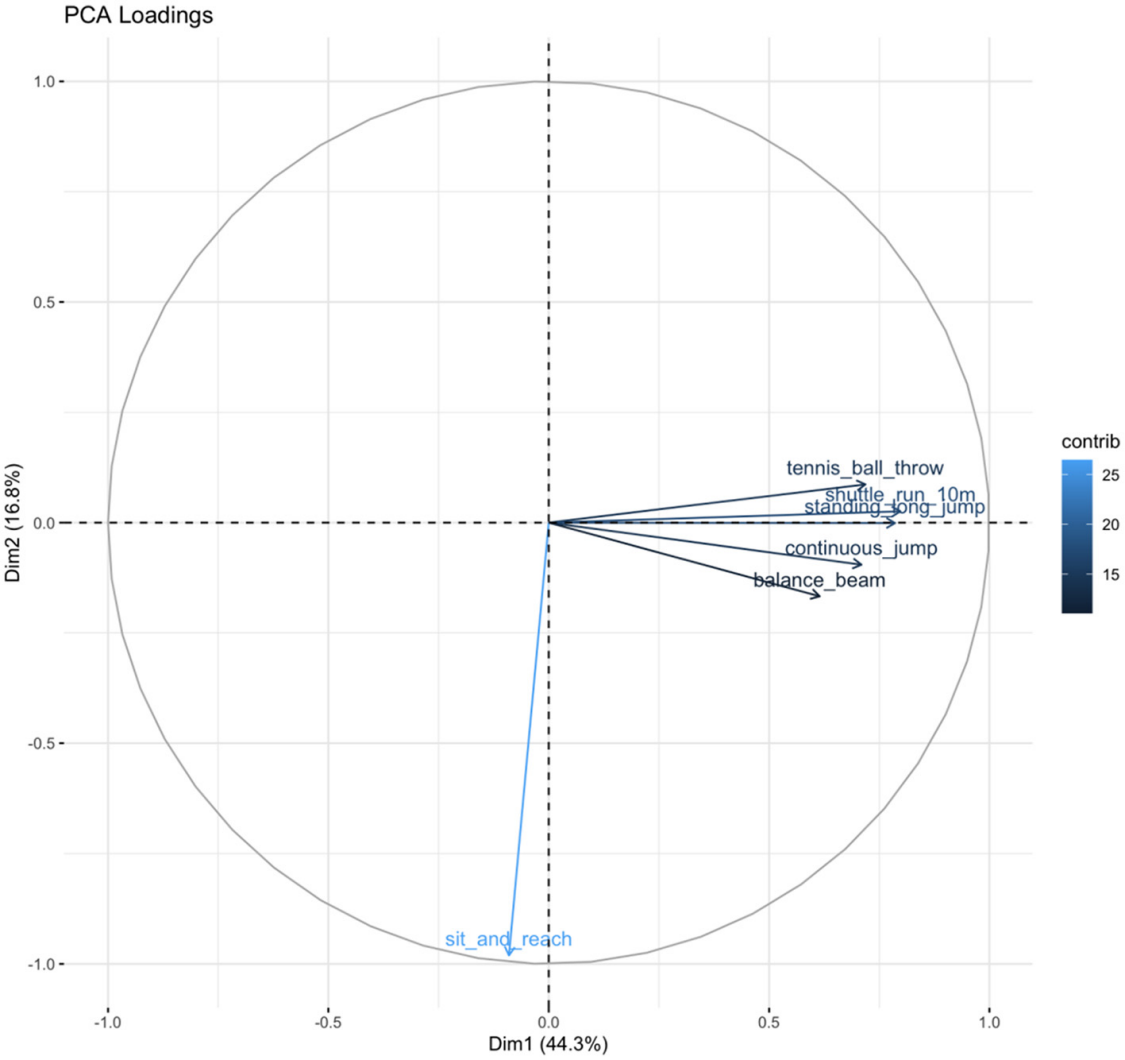
PCA loading.

K-means clustering on the six fitness Z-scores yielded a stable 3-cluster solution (Figures 1, 2). The clusters were characterized as: Cluster 1 (Low Fitness, *n* = 412), Cluster 2 (Moderate Fitness, *n* = 1,200), and Cluster 3 (High Fitness, *n* = 1,568) (Table 2). The clustering solution explained 39.3% of the total variance (R^2^ = 0.393). Principal Component Analysis (PCA) for visualization showed clear separation of the three clusters in the space of the first two principal components (cumulative variance explained = 61.1%) (Figures 3, 4).

**Table 2:** Physical fitness cluster characteristics.

**Cluster**	***n***	**Standing long jump**	**Tennis ball throw**	**Sit and reach**	**10 m shuttle run**	**Balance beam**	**Continuous jump**
1	412	−1.24 ± 0.62	−1.00 ± 0.54	0.13 ± 0.87	−1.58 ± 1.12	−2.65 ± 1.25	−1.60 ± 1.47
2	1,200	−1.04 ± 0.58	−0.84 ± 0.51	0.28 ± 0.89	−0.99 ± 0.77	−0.22 ± 0.59	−0.83 ± 0.96
3	1,568	0.00 ± 0.64	−0.09 ± 0.72	0.02 ± 0.94	0.13 ± 0.52	0.15 ± 0.61	0.25 ± 0.51

### 3.3 Association between fitness phenotypes and body shape

All nine body shape indicators differed significantly across the three clusters (all *p* < 0.001). Standard ANOVA was used for height, sit height, weight, chest circumference, hip circumference, shoulder breadth, and foot length. Welch's ANOVA was applied for waist circumference and pelvic width due to significant heteroscedasticity (Levene's test *p* < 0.05). As shown in Table 3, a clear gradient was observed: the High Fitness cluster had the most developed body shape metrics, followed by the Moderate and Low Fitness clusters. Effect sizes (Cohen's f) ranged from small (0.025 for waist circumference) to medium-large (0.190 for height).

**Table 3:** Comparison of body shape indicators across physical fitness clusters.

**Indicator**	**Cluster**	**Mean ±SD**	***F***	**Cohen**	***p***
Height (cm)	1	98.10 ± 4.42	371.971	0.190	< 0.001
	2	100.25 ± 4.76			
	3	105.24 ± 5.01			
Sit height (cm)	1	56.30 ± 2.50	237.131	0.130	< 0.001
	2	57.37 ± 2.63			
	3	59.61 ± 2.93			
Weight (kg)	1	14.92 ± 2.07	155.278	0.125	< 0.001
	2	15.59 ± 2.16			
	3	17.16 ± 2.55			
Chest circumference (cm)	1	51.46 ± 2.86	63.433	0.089	< 0.001
	2	51.85 ± 2.76			
	3	53.15 ± 3.06			
Waist (cm)	1	48.40 ± 3.56	38.543	0.025	< 0.001
	2	48.40 ± 3.56			
	3	49.84 ± 4.02			
Hip circumference (cm)	1	53.28 ± 3.92	87.505	0.052	< 0.001
	2	53.81 ± 3.87			
	3	55.96 ± 4.21			
Shoulder breadth (cm)	1	21.94 ± 1.41	121.192	0.071	< 0.001
	2	22.30 ± 1.40			
	3	23.12 ± 1.41			
Width of pelvic (cm)	1	16.38 ± 1.27	60.978	0.132	< 0.001
	2	16.40 ± 1.23			
	3	16.96 ± 1.15			
Foot length (cm)	1	15.53 ± 1.23	152.711	0.088	< 0.001
	2	15.77 ± 1.01			
	3	16.50 ± 1.05			

(Table content as per original, with added 'Test' column specifying ANOVA/Welch and 'Cohen's f' column).

### 3.4 Development of the composite body shape index

Multivariate logistic regression (low fitness cluster as reference) identified four significant predictors: height (β = −1.156, *p* < 0.001), weight (β = −0.412, *p* = 0.028), waist circumference (β = 0.493, *p* < 0.001), and pelvic width (β = 0.225, *p* = 0.004). The derived body shape index was:

BSI = −2.376 – 1.156 × Height (cm) – 0.412 × Weight (kg) + 0.493 × Waist Circumference (cm) + 0.225 × Pelvic Width (cm). Higher values of waist circumference and pelvic width increased the odds of low fitness, while greater height and weight decreased it. The model showed no significant multicollinearity (all VIFs < 2.5) and adequate fit (Hosmer-Lemeshow test *p* = 0.553) (Table 4).

**Table 4:** Overall summary of the logistic regression model for predicting poor physical fitness risk.

**Predictor**	**β co-efficient (SE)**	**Odds ratio (OR)**	**95% CI**	***p*-value**
Height (cm)	−1.156 (0.215)	0.315	0.207–0.480	< 0.001
Weight (kg)	−0.412 (0.187)	0.662	0.459–0.955	0.028
Waist circumference (cm)	0.493 (0.098)	1.637	1.351–1.983	< 0.001
Pelvic width (cm)	0.225 (0.078)	1.252	1.075–1.459	0.004
Hosmer-Lemeshow: χ^2^ = 6.85, *p* = 0.553, R^2^ = 0.065				

### 3.5 Predictive performance and validation

**Figure 5:**
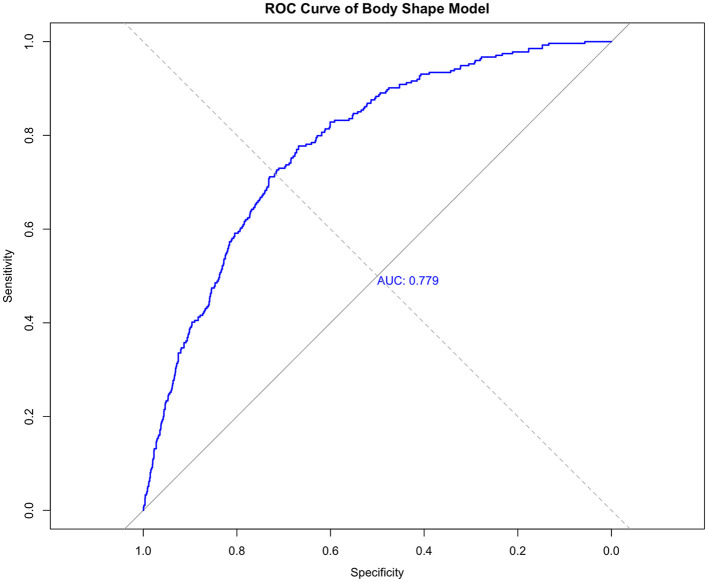
Receiver operating characteristic (ROC) curve for the composite body shape index in predicting the low physical fitness phenotype.

**Figure 6:**
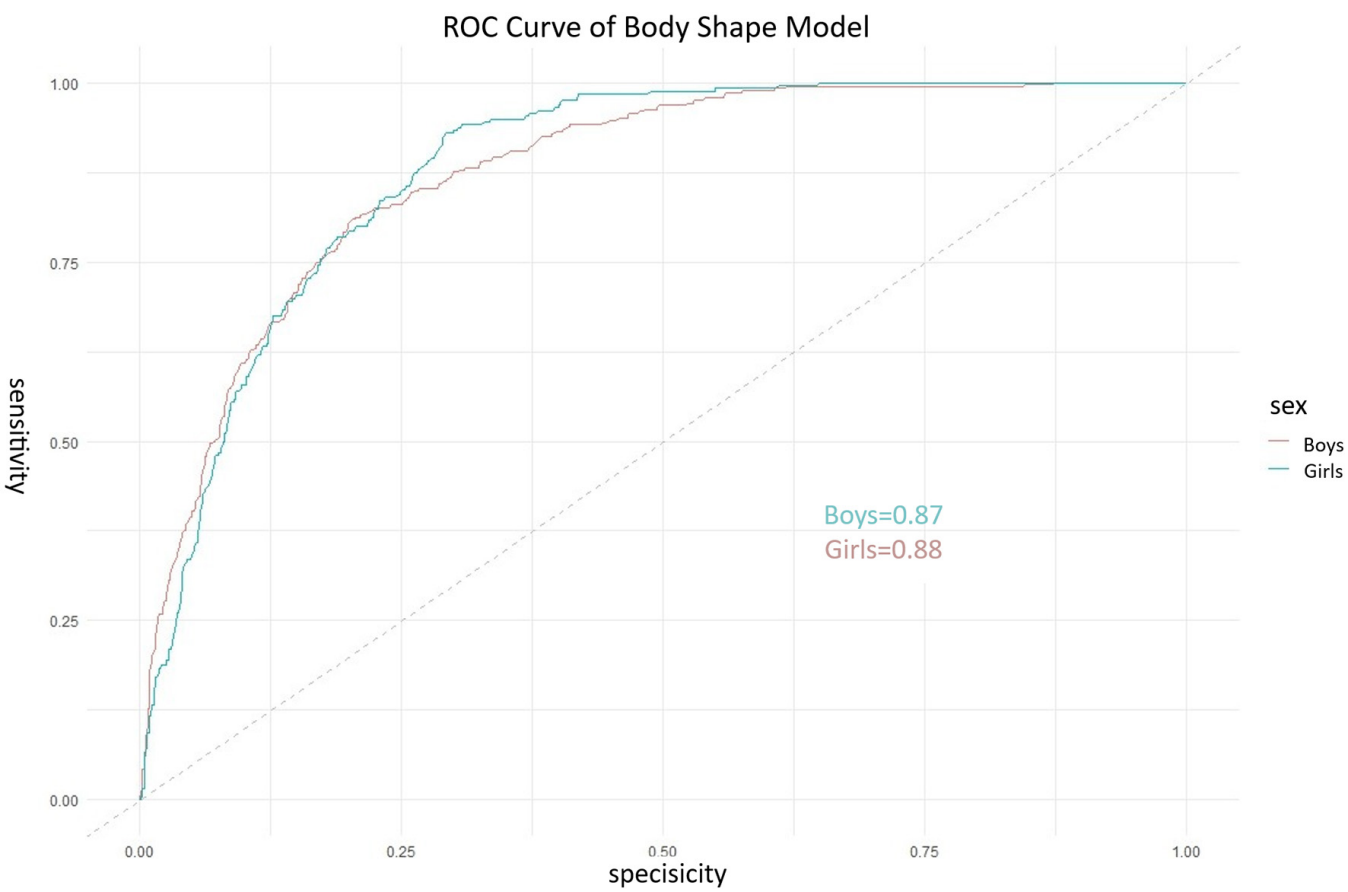
Receiver operating characteristic (ROC) curves of the composite Body Shape Index for predicting low physical fitness risk, stratified by sex. The solid line represents the ROC curve for boys (AUC = 0.87), and the dashed line represents the ROC curve for girls (AUC = 0.88). The diagonal gray line indicates the reference line of no discrimination (AUC = 0.50). The close and high AUC values in both sexes demonstrate the strong and consistent predictive ability of the BSI across genders in this population.

The ROC curve for the BSI is shown in Figure 5. The apparent AUC was 0.779 (95% CI: 0.742–0.800). The 10-fold cross-validated mean AUC was 0.765 (SD = 0.021), indicating robust internal validity. At the optimal cut-off score of −2.003, sensitivity was 77.7% and specificity was 66.8%. Further analysis regarding gender differences revealed that the composite body shape index demonstrated good predictive performance in both boys and girls. In the subsample of boys, the area under the curve (AUC) of the BSI for predicting low physical fitness risk was 0.87 (95% CI: 0.84–0.90), while in the subsample of girls, the AUC was 0.88 (95% CI: 0.85–0.91). These results suggest that within the current preschool-aged population of 3–6 years, the body shape index developed in this study shows consistent applicability and strong predictive ability across sexes. Nevertheless, if sex-specific models were to be constructed separately, the regression coefficients might differ, which presents a potential direction for refinement in future research. The comparative analysis and sex-stratified results are visually presented in Figure 6 through ROC curves.

### 3.6 Comparison with BMI and sex-stratified analysis

**Figure 7:**
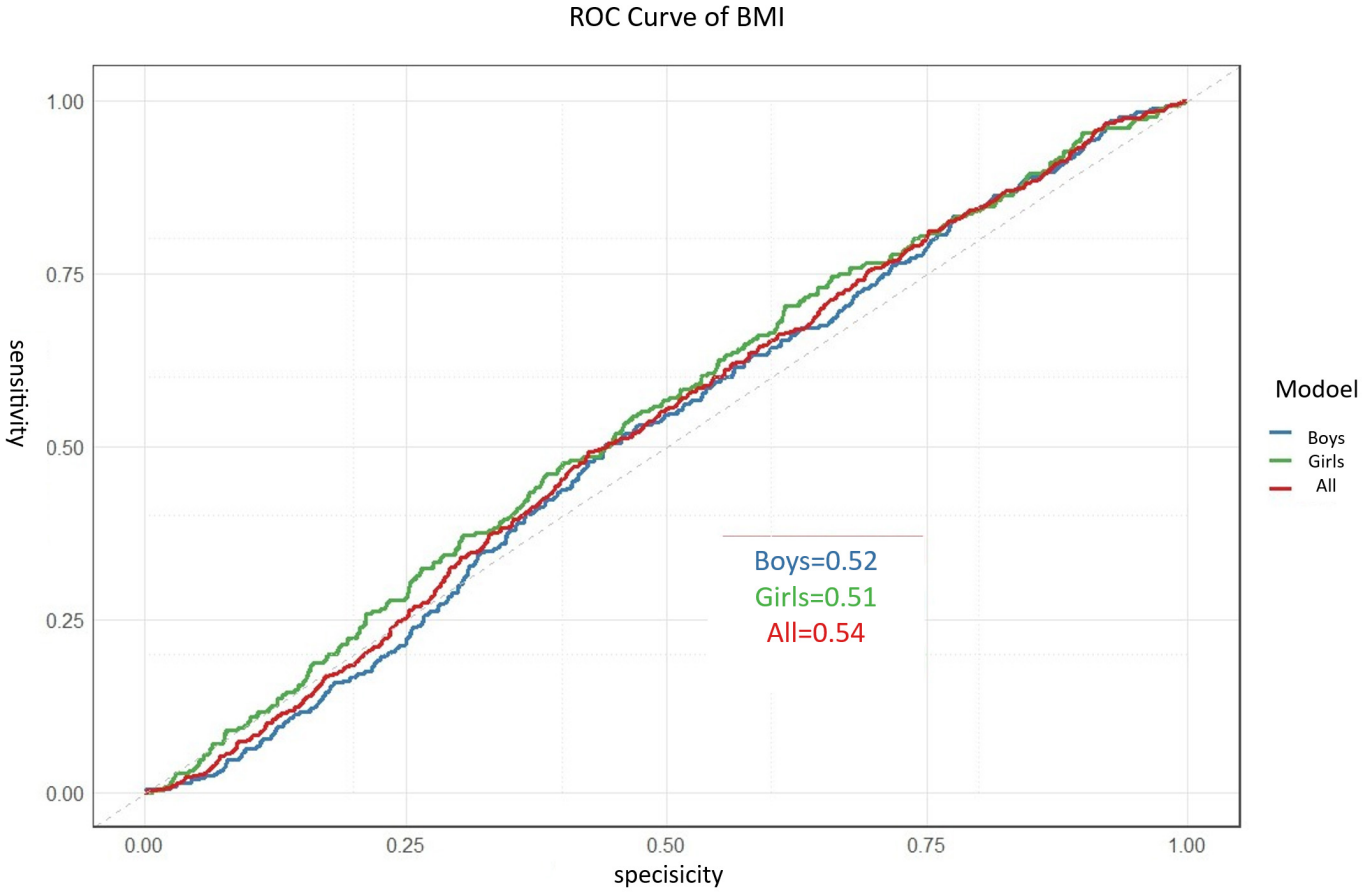
Receiver operating characteristic (ROC) curves of BMI for predicting low physical fitness phenotype in preschool children. Results are shown for the total sample and stratified by sex. The AUC (95% Confidence Interval) values were 0.54 for the total sample, 0.52 for boys, and 0.51 (0.46–0.56) for girls. The diagonal gray line indicates the reference line of no discrimination (AUC = 0.5).

To validate the superiority of the constructed Body Shape Index (BSI), we conducted a direct comparison with the traditional BMI. Separate logistic regression models, using only age- and sex-standardized BMI as the predictor, were fitted for boys, girls, and the combined sample to predict membership in the low physical fitness phenotype. The discriminative performance of BMI was limited in all groups. The area under the curve (AUC) was 0.52 (95% CI: 0.47–0.57) for boys, 0.51 (95% CI: 0.46–0.56) for girls, and 0.54 (95% CI: 0.50–0.58) for the total sample (Figure 7). These results indicate that BMI alone has only marginal discriminative ability, with AUC values not meaningfully exceeding the chance level of 0.5, in identifying children at risk of low physical fitness in this study population.

To formally test the difference in diagnostic performance between the composite BSI and the BMI-only model, we performed DeLong's test for two correlated ROC curves using the total sample. The results are summarized in Table 5. The BSI demonstrated a significantly larger AUC (0.779) compared to the BMI-only model for the total sample (0.54), with a difference of 0.239 (Z = 10.15, *p* < 0.001). This provides statistical confirmation of the superior discriminant validity of the proposed body shape index over the traditional BMI metric.

**Table 5:** Comparison of predictive performance between BSI and BMI using DeLong‘s Test.

**Model**	**AUC (95% CI)**	**Sensitivity**	**Specificity**	**DeLong‘s test (vs. BSI)**
Body shape index (BSI)	0.779 (0.742–0.800)	77.7%	66.8%	Reference
BMI-only (Total sample)	0.54 (0.50–0.58)	61.5%	58.3%	Z = 10.15, *p* < 0.001

AUC, Area Under the Curve; CI, Confidence Interval. Sensitivity and specificity are reported at the optimal cut-off point (Youden's index) for each model. The *p*-value indicates the significance of the difference in AUC between the two models.

## 4 Discussion

This study achieved its 3-fold aim. First, using a data-driven clustering approach with enhanced methodological reporting, we identified three distinct, naturally emerging physical fitness phenotypes among preschool children. Second, we established strong gradient associations between these phenotypes and a range of body shape indicators. Third, and most innovatively, we developed and validated a composite Body Shape Index (BSI) that demonstrates good performance in screening for low fitness risk, with statistical evidence supporting its discriminant advantage over the traditional BMI metric in our sample.

The core methodological contribution lies in the sequential application of unsupervised learning (for phenotype discovery) and supervised learning (for index development), bridging the gap between holistic pattern identification and practical screening tool creation. The gradient in body shape across fitness clusters underscores the integrated nature of physical development. The logistic regression model provides a quantitative link, revealing that after accounting for overall body size (height and weight), specific circumferential, and skeletal breadth measures (waist circumference and pelvic width) are independently associated with fitness risk (17). This suggests that body proportions and fat distribution may be more sensitive morphological correlates of integrated fitness in young children than body size alone (18, 19), which aligns with and extends critiques of BMI, as it does not differentiate these dimensions (20, 21).

Compared to prior work focusing on single test correlations or long-term growth indices, our study shifts the application scenario toward immediate, cross-sectional risk screening (16, 22, 23). The BSI's discriminative ability (AUC = 0.779) and its statistically significant superiority over BMI (AUC = 0.54; *p* < 0.001 per DeLong's test) highlight its potential as a more informative morphological indicator in this context. Furthermore, the sex-stratified analysis revealed that the BSI performed consistently well for both boys (AUC = 0.87) and girls (AUC = 0.88), supporting its internal generalizability within the preschool age range of our study population. Taken together, these findings suggest that the BSI, based on simple anthropometric measures, could serve as a promising preliminary screening tool to identify children who may benefit from more comprehensive fitness assessments.

However, our interpretations are tempered by the study's design and methodological choices. We acknowledge that the cross-sectional data preclude causal inferences between morphology and fitness phenotypes. Furthermore, the decision not to include age, sex, and survey year as covariates in the final predictive model was based on statistical and conceptual grounds (i.e., prior control via Z-score standardization for age/sex, and non-significance of survey year), which we argue prevents over-adjustment. Nonetheless, this choice, along with the potential for unmeasured cohort effects from combining survey waves, should be considered when interpreting the model's coefficients and generalizing findings. Critically, the external validity of the BSI requires confirmation in independent and diverse populations, and its predictive utility over time warrants longitudinal investigation before it can be recommended for clinical or public health practice. The current findings primarily offer a methodological advancement and a proof-of-concept for an integrated, morphology-based screening approach.

## 5 Conclusion

This study identified three distinct physical fitness phenotypes among preschool children using a data-driven clustering approach. The derived composite Body Shape Index (BSI), integrating height, weight, waist circumference, and pelvic width, demonstrated good and consistent discriminative ability for identifying children at risk of low fitness (AUC = 0.779), significantly outperforming BMI alone. While promising as a practical screening tool for rapid risk stratification during routine health checks, its cross-sectional design and single-population origin necessitate future longitudinal and external validation. The methodological integration of unsupervised and supervised learning offers a novel framework for translating holistic fitness patterns into actionable screening indices.

## Data Availability

The raw data supporting the conclusions of this article will be made available by the authors, without undue reservation.
